# Dimensions of role and identity in young informal workers in the tourism sector

**DOI:** 10.1192/j.eurpsy.2023.2029

**Published:** 2023-07-19

**Authors:** K. J. Obispo Salazar

**Affiliations:** Programa de Psicología, Universidad del Magdalena, Santa Marta, Colombia

## Abstract

**Introduction:**

Work is a *sine qua non* condition of “normal” life for most people, since it is the main source of income, feelings and social integration (Méda, 2007). It is an important factor of socialization, as well as an identity provider (Agulló, 1998). Identity was assumed as a constant construction process in which the person is positioned and recognized, and which has an important relationship with the dimensions of the role (Scheibe, 1995). All this in a context of insertion into the labor market for young people, which is usually framed in informality.

**Objectives:**

Describe the dimensions of the role and identity of the young informal worker in the tourism sector of Santa Marta

**Methods:**

This was a qualitative study, with a phenomenological design. The participants were selected for convenience, and the sample size was determined by the saturation criterion. A total of nine young informal workers participated. The semi-structured interview and the content analysis technique were used for data analysis.

**Results:**

The dimensions of status, involvement and assessment allowed us to deduce that the role played by young people was central in the description and construction of their identity, as well as the implications and the place occupied by the tourist, the family and co-workers in the activity that they carry out. develop, because they are the ones who validate and motivate people to stay and mobilize in that work context, expressing *“So I tried when the tourist managed to capture my attention I started explaining about the…”* (P4). That these dimensions are high indicates that there is a close relationship between role and identity.

**Conclusions:**

If identity is read from the social positions that are recognized by others (Scheibe, 1999), particularly the findings of this research showed that characters such as family, tourists, co-workers and friends intervene significantly in the recognition of the roles assumed, which makes the young person stay in this activity and market, as well as find satisfaction in it. Through the dimensions of the role, it was evidenced that at work it is possible to configure the identity of young people. For Lucena et al. (2018) when a person who does part of this type of work and refers to it, is talking about himself.

**Disclosure of Interest:**

None Declared

